# Mitochondrial Vulnerability and Increased Susceptibility to Nutrient-Induced Cytotoxicity in Fibroblasts from Leigh Syndrome French Canadian Patients

**DOI:** 10.1371/journal.pone.0120767

**Published:** 2015-04-02

**Authors:** Yan Burelle, Chantal Bemeur, Marie-Eve Rivard, Julie Thompson Legault, Gabrielle Boucher, LSFC Consortium, Charles Morin, Lise Coderre, Christine Des Rosiers

**Affiliations:** 1 Faculty of Pharmacy, Université de Montréal, Montréal, Canada; 2 Montreal Heart Institute, Montreal, Canada; 3 Department of Nutrition, Faculty of Medicine, Université de Montréal, Montreal, Canada; 4 Department of Pediatrics and Clinical Research Unit, Complexe hospitalier de la Sagamie, Chicoutimi, QC, Canada; 5 Department of Medicine, Faculty of Medicine, Université de Montréal, Montréal, Canada; Instituto de Investigación Hospital 12 de Octubre, SPAIN

## Abstract

Mutations in LRPPRC are responsible for the French Canadian variant of Leigh Syndrome (LSFC), a severe disorder characterized biochemically by a tissue-specific deficiency of cytochrome c oxidase (COX) and clinically by the occurrence of severe and deadly acidotic crises. Factors that precipitate these crises remain unclear. To better understand the physiopathology and identify potential treatments, we performed a comprehensive analysis of mitochondrial function in LSFC and control fibroblasts. Furthermore, we have used this cell-based model to screen for conditions that promote premature cell death in LSFC cells and test the protective effect of ten interventions targeting well-defined aspects of mitochondrial function. We show that, despite maintaining normal ATP levels, LSFC fibroblasts present several mitochondrial functional abnormalities under normal baseline conditions, which likely impair their capacity to respond to stress. This includes mitochondrial network fragmentation, impaired oxidative phosphorylation capacity, lower membrane potential, increased sensitivity to Ca^2+^-induced permeability transition, but no changes in reactive oxygen species production. We also show that LSFC fibroblasts display enhanced susceptibility to cell death when exposed to palmitate, an effect that is potentiated by high lactate, while high glucose or acidosis alone or in combination were neutral. Furthermore, we demonstrate that compounds that are known to promote flux through the electron transport chain independent of phosphorylation (methylene blue, dinitrophenol), or modulate fatty acid (L-carnitine) or Krebs cycle metabolism (propionate) are protective, while antioxidants (idebenone, N-acetyl cysteine, resveratrol) exacerbate palmitate plus lactate-induced cell death. Collectively, beyond highlighting multiple alterations in mitochondrial function and increased susceptibility to nutrient-induced cytotoxicity in LSFC fibroblasts, these results raise questions about the nature of the diets, particularly excess fat intake, as well as on the use of antioxidants in patients with LSFC and, possibly, other COX defects.

## Introduction

The French Canadian variant of Leigh Syndrome (LSFC) is an autosomal recessive mitochondrial respiratory chain disorder with a carrier frequency of about 1/23 in the Saguenay-Lac-St-Jean region of Quebec [1–3]. It is caused by mutation of the *LRPPRC* gene encoding a leucine-rich pentatricopeptide repeat protein that regulates the stability of most mitochondrial mRNA’s, all of which encode proteins involved in oxidative phosphorylation (OXPHOS) [4,5]. Most patients examined to date are homozygous for a single missense mutation predicting a A354V substitution, which results in low steady state levels of a mutated LRPPRC protein in all tissues, and a defect in the translation of mtDNA-encoded subunits affecting complex IV of the electron transport chain (ETC) or cytochrome c oxidase (COX) subunits preferentially [6]. Biochemically, LSFC is characterized primarily by a severe decrease in COX activity in the brain and liver, while in other tissues, such as kidneys, skeletal muscle, and heart, COX activity is affected to a lesser extent (50–80% residual activity) [1,3]. Recently, tissue-specific responses to the *LRPPRC* mutation were also reported for the protein abundance of other complexes of the ETC [7]. Clinically, patients present developmental delay, hypomorphism, characteristic facial appearance, and chronic moderate hyperlactatemia. In addition, LSFC is distinguished from classic Leigh syndrome by the occurrence of fulminant acidotic crises, which represent the major cause of morbidity in these patients [2,3].

Despite significant advances in our understanding of the molecular genetics of LSFC, the pathogenic mechanisms underlying this severe and unpredictable disease currently remain unclear [4–6]. Moreover, treatment strategies for these patients are non-existent. This is in part due to a lack of data on the impact of the LRPPRC A354V mutation on the various facets of mitochondrial function. As in most mitochondrial diseases, impaired capacity to generate ATP is often believed to be the main culprit. However, mitochondria also play a central role in numerous other vital processes, including Ca^2+^ dynamics, production of reactive oxygen species (ROS), regulation of redox state, and triggering of programmed cell death, all of which could contribute to dysfunction and death, particularly when cells are faced with stressful conditions [8–10].

A major unresolved question in LSFC relates to the mechanisms leading to acidotic crises, which probably mark the transition from compensated COX deficiency to irreversible neurological damage and multiple organ failure. Clinical observations suggest that crises often develop during, or shortly after, exposure to various types of stress, including infectious/inflammatory states, emotional shocks, and excess of nutrients [3]. However, specific factors or conditions that play an important role in the development and worsening of crises still remain to be identified.

In the present study, we have performed a detailed characterization of the morphological and functional phenotype of mitochondria in skin fibroblasts from control subjects and LSFC patients. In addition, we have used these cells as a working model to both identify factors triggering premature cell death and test the protective effect of compounds targeting well-defined aspects of mitochondrial function. Our main hypotheses were that LSFC fibroblasts *i*) would display multiple mitochondrial abnormalities that increase vulnerability to stress, *ii*) would be more sensitive to cell death when exposed to one or several factors relevant to acidotic crises, namely proinflammatory cytokines, stress hormones, low pH, or excess nutrients, and *iii*) would be protected by treatments targeting potential consequences of COX deficiency such as impaired ETC flux, oxidative stress, mitochondrial cell death signaling, and accumulation of toxic metabolites.

## Method

### Cell culture

The protocol was approved by the Research Ethics Committees of the Centre de santé et de services sociaux de Chicoutimi, the Montreal Heart Institute, the McGill University Research Center and the Université de Montréal. Skin biopsies were obtained from subjects after receiving written informed consent from either the subject or their legal guardian(s). Human skin fibroblasts from three LFSC patients and four control subjects, aged between 8 and 39 years, were obtained from the LSFC Consortium Biobank (Université du Québec à Chicoutimi, Chicoutimi, QC, Canada) and McGill University Research Center (Table 1). All of these LSFC patients exhibited the C1119T substitution (A354V). Cells were grown at 37°C and 5% CO_2_ in high glucose (4.5 g/L) Dulbecco's modified Eagle's medium (DMEM) (Wisent Inc, St-Bruno, QC, Canada) supplemented with 10% fetal bovine serum (FBS; Invitrogen, Carlsbad, CA, USA), MEM vitamins and nonessential amino acids solutions (Mediatech, Manassa, VA, USA), penicillin (100 U/ml) and streptomycin (10 mg/ml). Unless stated, all chemicals were obtained from Sigma-Aldrich (St-Louis, MO, USA). All experiments were performed in primary cells between passages 5 and 15, except for those evaluating “mitochondrial function and network morphology” and “immunocytochemistry”. For the latter experiments, which require larger amounts of material, fibroblasts were immortalized by transduction with retroviral vectors expressing the HPV-16 E7 gene, and the catalytic component of human telomerase, as described [11]. A previous study has demonstrated that mitochondrial dysfunctions in primary cells from patients with genetic respiratory chain defects are preserved after immortalization [12].

**Table 1:** Control and Leigh Syndrome French Canadian (LSFC) skin fibroblast cell lines.

Category	Cell lines	Sex	Age (years)
**Control**	**EBS-3**	**Female**	**9**
**Control**	**EBS-4**	**Female**	**5**
**Control**	**JGE**	**Male**	**39**
**Control**	**MCO**	**Female**	**19**
**LSFC**	**AL002**	**Male**	**25**
**LSFC**	**AL005**	**Female**	**25**
**LFSC**	**AL006**	**Female**	**8**

### Cell treatments

The effects of various stress factors relevant to acidotic crises on cell death were assessed using caspase 3/7 activities and lactate dehydrogenase (LDH) release as markers. Briefly, cells were plated at a density of 10,000 cells/well in six well plates (85–90% confluence) in the above mentioned culture medium for 4 h. They were then exposed for either 24 h (caspase 3/7 activities) and 48 h (LDH release) to the following stress factors in a serum-free non-supplemented DMEM medium: high glucose (30 mM), high lactate (10 mM), low pH (7 mM H_2_CO_3;_ pH 7.0), TNFα (100 ng/mL), isoproterenol (10 μM), bovine serum albumin (BSA)-conjugated palmitate (1.3% BSA, 1 mM total palmitate), or H_2_O_2_ (100 μM). The latter condition was used as a positive control. For further characterization of the impact of palmitate plus lactate (PL), LDH release was assessed after 34 h; this time corresponds to 50% of maximal LDH release, therefore optimizing the opportunity to see significant changes after treatments. Cells were incubated in presence of the following agents added simultaneously with PL: methylene blue (125, 250, 500 nM [13]), resveratrol (10, 50 and 100 μM [14]), N-acetyl cysteine (1 mM, [15]), idebenone (10 and 25 μM, [16]), sildenafil (0.3, 1 and 10 μM, [17–19]), cyclosporin-A (0.2 μM, [20,21]), fenofibrate (20 μM, [22]), L-carnitine (1, 3 and 10 mM, [23]), dinitrophenol (10, 25 and 50 μM, [24]), or sodium propionate (0.2 mM, [25,26]). These concentrations were chosen based on published literature describing the protective effect of these compounds. Experiments were first performed in triplicates using 1 control and 1 LSFC cell line, and then replicated in a larger number of cell lines for compounds that reduce cytotoxicity, as indicated in the Figure Legend.

### Mitochondrial functions and network morphology

#### Respiratory function

Respiration was measured polarographically using Clark-type electrodes (Hansatech Instruments, Norfolk, UK) [27]. Five million fibroblasts were permeabilized with digitonin (10 μg/ml) for 2.5 min and immediately incubated at 30°C under continuous stirring in 500 μl of a respirometry buffer containing (in mM): 10 Tris-MOPS, pH 7.4, 125 KCl, 10 KH_2_PO_4_, 0.05 EGTA, 2.5 MgCl_2_, 1 ADP, and 2 mg/ml BSA. Baseline respiration was recorded in the absence of respiratory substrates, after which 5 mM glutamate + 2.5 mM malate or 5 mM succinate + 1 μM rotenone was added to evaluate maximal ADP-stimulated respiration with complex I and complex II donors, respectively. At the end of each test, a complex I (1 μM rotenone) or a complex II (500 μM malonate) inhibitor was added in order to correct for non-mitochondrial O_2_ consumption. Rates of respiration were expressed in μmoles of O_2_ per minute per million cells. These measurements were performed in one LSFC cell line (AL-006) and its sex- and age-matched control counterpart (EBS-4) and these results were confirmed in another pair of sex and age-matched cell lines (EBS-3 and AL-002).

#### Ca2+-induced opening of the permeability transition pore (PTP)

Susceptibility to Ca^2+^-induced opening of the permeability transition pore (PTP) was determined as previously described with minor modifications [27]. Briefly, 5 millions of permeabilized fibroblasts were incubated at 25°C in 600 μL of a buffer containing (in mM): 250 sucrose, 10 Tris-MOPS, pH 7.4, 0.005 EGTA, 1 KH_2_PO_4_, supplemented with either 5 mM glutamate + 2.5 mM malate or 5 mM succinate + 1 μM rotenone. Changes in extra-mitochondrial calcium concentration were monitored on a Hitachi F4500 fluorescence spectrometer using Calcium-green 5N (1 μM, excitation/emission: 505/535 nm). Residual calcium concentration was adjusted to the same level at the beginning of every experiment by adding a small amount of EGTA. Calcium pulses (83 nmoles/pulse) were added at 2 min intervals until a Ca^2+^-induced Ca^2+^-release was observed. Calcium retention capacity (CRC) was taken as the total amount of Ca^2+^ accumulated by mitochondria prior to the Ca^2+^ pulse triggering PTP-dependent Ca^2+^ release. These measurements were performed in one control (EBS-4) and one LSFC (AL-006) cell line and these results were confirmed in another pair of cell lines (EBS-3 and AL-002).

#### Mitochondrial membrane potential

Mitochondrial membrane potential was evaluated in intact cells by ratiometric imaging using dual staining with the membrane potential-dependent dye tetramethylrhodamine, ethyl ester (TMRE) and the membrane potential-independent dye MitoTracker Green (MTG) (Invitrogen, Burlington, ON, Canada) [28]. One advantage of this ratiometric method is that it takes into account differences in TMRE fluorescence attributable to variation in mitochondrial content between experimental groups and/or focal plane between each image [28]. Fibroblasts cultured on 35 mm glass-bottom petri dishes (MatTek Corporation, Ashland, MA, USA) were loaded with 125 nM MTG and 8 nM TMRE for 45 min at 37°C. Fibroblasts were then washed three times with a MTG-free buffer prior to imaging. TMRE was left in the buffer to limit artefacts linked to progressive loss of cellular TMRE. Images were taken on a Nikon TE2000 inverted epifluorescence microscope with a 40X CFI Plan Fluor oil immersion objective (N.A. 1.30; W.D. 0.2 mm) using standard TRITC and FITC filter cube sets. Light intensity (12% of lamp output) and exposure time (100 ms) were kept to a minimum to limit phototoxicity. Mean TMRE and MTG fluorescence intensity were analyzed using the Simple PCI software (Hamamatsu Photonics, Bridgewater, NJ, USA). A minimum of ~50 cells per dish were analyzed. Membrane potential was reported as the ratio TMRE/MTG. Similar results were also obtained using TMRE fluorescence alone. These measurements were performed in one control (EBS-3) and one LSFC (AL-002) cell line. Importantly, since in some experimental conditions, MTG fluorescence was reported to be somewhat sensitive to changes in mitochondrial membrane potential [29], preliminary experiments using the uncoupler CCCP were performed to confirm that under the loading conditions used in the present study, MTG fluorescence was independent of membrane potential. It should also be noted that in our studies similar results were obtained for membrane potential when TMRE fluorescence was used alone. Mitochondrial membrane potential was also assessed by quantifying cellular uptake of the potentiometric probe rhodamine123 (Rh123: Invitrogen, Burlington, ON, Canada). Briefly, fibroblasts (150,000) were incubated for 120 min at 37°C with 10 μg/ml Rh123. Following washout of the extracellular probe, cellular Rh123 was extracted by lysing cells in 1 mL of butanol. Rh123 fluorescence in butanol extracts was determined (excitation/emission: 508/536 nm) on a Tecan Safire plate reader. Cellular uptake of Rh123 was then calculated from a standard curve generated using known concentrations of Rh123. These confirmatory measurements were performed in one control (EBS-4) and one LSFC (AL-006) cell line.

#### Mitochondrial superoxide production

Fibroblasts grown on 35 mm glass-bottom petri dishes were loaded with 5 μM MitoSox Red (Invitrogen, Burlington, ON, Canada) for 20 min at 37°C. Following washout of the extracellular probe, cells were imaged as described above using a HQ500/30x Q530LP HQ610/75m cube set (Chroma Technology Group, Rockingham, VT, USA). Mean MitoSox Red fluorescence intensity was analyzed in a minimum of ~50 cells per dish. These measurements were performed in one control (EBS-4) and one LSFC (AL-006) cell line.

#### Mitochondrial network morphology

Fibroblasts grown on 35 mm glass-bottom petri dishes were incubated with 0.5 μM MTG for 20 min at 37°C. Following washout of extracellular MTG, cells were placed at 37°C on the stage of a Zeiss LSM 510 confocal microscope (Carl Zeiss, Oberkochn, Germany) equipped with a 63X oil immersion objective. MTG was excited using the 488 nm argon laser and emission was recorded through a 500–550 band pass filter. Quantitative analysis of mitochondrial morphology was conducted using the morphometric analysis application of the ImageJ software (Rasband WS., U.S. National Institutes of Health, Bethesda, MD, USA, imagej.nih.gov/ij/, 1997–2012). Images of the mitochondrial network were first denoised with a median filter, followed by a top hat filter, to enhance the separation of mitochondria from the background [30,31]. Images where then binarized to create a mask, and submitted to particle analysis for acquiring Form Factor (FF = 4π*Area/perimeter^2^) and Aspect Ratio (AR = long axis/short axis) values. FF corresponds to the degree of mitochondrial branching, whereas AR provides information about mitochondrial length [30]. An FF value of 1 corresponds to a circular, unbranched mitochondrion, while higher FF values indicate a longer, more-branched mitochondrion. An AR value of 1 indicates a perfect circle and, as mitochondria elongates and become more elliptical, AR increases. Morphological analyses were performed on a minimum of 10–12 cells per field of view. These measurements were performed in one control (EBS-4) and one LSFC (AL-006) cell line.

### Biochemical and molecular assays

#### Activities of citrate synthase (CS) and cytochrome c oxidase (COX)

Cells were resuspended for 35 s in a buffer containing (in mM): 20 MOPS pH 7.2, 3 EDTA, 250 sucrose, and 1 mg/ml digitonin, and centrifuged at 9,600 x g at 4ºC for 1 min. The cell pellet was then washed once before being resuspended in a digitonin-free buffer. The cells were then submitted to 3 freeze/thaw cycles and sonication for 1 min. Enzyme activities were measured using a Roche Cobas Fara spectrophotometer (Hoffman-La Roche, Bale, Switzerland) using previously established protocols [27]. CS activity was measured by monitoring the transfer of the CoA sulfhydryl groups to 5.5′-dithio-bis (2-nitrobenzoic acid) (DTNB). The assay was performed in buffer containing (in mM) 100 Tris-HCl, pH 8.0, 5 acetyl-CoA, 50 oxaloacetic acid, and 1.25 DTNB. The change of optical density was recorded at 412 nm for 150 s. COX activity was measured by monitoring the oxidation of reduced cytochrome *c* at 550 nm in buffer containing (in mM): 50 K_2_HPO_4_, pH 7.5, 0.6 cytochrome *c* and 69 sodium hydrosulfite. The change in optical density was recorded for 400 s at 37ºC. All enzyme activities were expressed in mU/min/mg of protein.

#### Lactate dehydrogenase release

LDH release was measured using the “CytoTox 96 NonRadioactive Cytotoxicity Assay” (Promega Corporation; Madison, WI, USA) according to the manufacturer’s instructions. Briefly, cells were plated in clear 96 well plates at a density of 10,000 cells per well. The cellular and supernatant LDH content was determined at 490 nm with a Synergy 2 Alpha Microplate Reader (Biotek, Winooski, VT, USA). Results were expressed as percent LDH in supernatant relative to total cellular LDH. All experiments were performed in triplicate.

#### Caspase 3/7 activity

Fibroblasts were plated in opaque 96 well plates at a density of 10,000 cells per well and caspase 3/7 activities were measured with the “Caspase-Glo 3/7 Assay Systems” (Promega Corporation; Madison, WI, USA) according to the manufacturer’s instructions. Luminescence was quantified with a Synergy 2 Alpha Microplate Reader. All experiments were performed in triplicate.

#### ATP levels

Fibroblasts were plated in opaque 96 well plates at a density of 10,000 cells per well and ATP content was measured with the ATPlite Luminescence Assay System according to the manufacturer's instruction (PerkinElmer, Waltham, MA, USA). Luminescence was quantified with a Synergy 2 Alpha Microplate Reader. All experiments were performed in triplicate.

#### Gel electrophoresis and immunoblotting

Fibroblasts were lysed in buffer containing (in mM): 25 Tris·HCl, pH 7.4, 150 NaCl, 1 sodium orthovanadate, 20 sodium fluoride, 10 sodium pyrophosphate, 2 EGTA, 2 EDTA, 1 phenylmethylsulfonyl fluoride, supplemented with 0.1% sodium dodecyl sulfate (SDS), 1.5% dodecyl-maltoside and a protease inhibitor cocktail [4]. The lysate was centrifuged for 30 min at 20,000 x g at 4°C to remove insoluble material, and the resulting supernatant was used for immunoblotting. Samples were electrophoresed on 8% SDS-polyacrylamide gel and transferred to nitrocellulose membranes. The membranes were blocked with 5% milk (wt/vol) in TBST (in mM: 25 Tris-HCl pH 7.6, 150 NaCl and 0.05% Tween 20) and incubated with the following primary antibodies: LRPPRC (1/10 000), VDAC (1/1000, Abcam), HSP60 (1/10 000, Abcam), actin (1/10 000, Abcam). LRPPRC polyclonal antibodies were raised in rabbit against a 22 amino acid peptide having the sequence CEPPESFEFYAQQLRKLRENSS (antibody 295–313; Zymed Laboratories, San Francisco, CA). After washing, membranes were incubated with the appropriate secondary antibodies conjugated to horseradish peroxidase. Antigen-antibody complexes were detected by the enhanced chemiluminescence method. Quantitative analysis was performed with a GS-800 calibrated densitometer (Bio-Rad) using Quantity One 1-D Analysis Software (Bio-Rad Laboratories, Inc. Hercules, CA, USA).

#### Immunocytochemistry

Fibroblasts were grown on glass bottom culture dishes (MatTek Corporation, Ashland, MA, USA), washed with PBS and fixed in 2% paraformaldehyde for 30 min at room temperature (RT). Fixed cells were permeabilized with PBS + 0.1% Triton for 10 min at RT and then blocked using PBS + 10% FBS for 30 min at RT. The cells were then incubated with the following primary antibodies in PBS + 5% FBS for 1 h at RT: LRPPRC (1/500), pyruvate dehydrogenase (PDH; Abcam, 1/2000). After three 5 min washes with PBS + 5% FBS, cells were incubated with the following secondary antibodies in PBS + 5% FBS for 1 h at RT: anti-rabbit AlexaFluor 488 and anti-mouse AlexaFluor 594 (Invitrogen, 1/1000). Cells were then washed three times (5 min each) in PBS. Slides were mounted with Prolong Gold antifade reagent containing the nucleus specific staining DAPI (4',6-Diamidino-2-Phenylindole, Invitrogen). Immunocytofluorescence was viewed on a Zeiss LSM 510 confocal fluorescence microscope (Carl Zeiss, Oberkochen, Germany) and image analysis was performed using Zen 2009 Light Edition software.

### Statistical analysis

Results are expressed as mean ± standard error of the mean (SE). Normality of data was tested using: (i) Shapiro-Wilk for t-test analysis, except for LRPPRC and COX for which the test cannot be applied since n<5 (n = 4); and (ii) z-score (Y-mean/SD) for each individual datum (Y) followed by the Agostino-Pearson Omnibus Normality test for analysis of variance (ANOVA). All data tested were normally distributed. When one control and one patient cell lines were compared, a paired t-test or a two-way ANOVA for repeated measures were performed for comparison of these particular cell lines. A Bonferroni correction for multiple testing was applied. These analyses were done using GraphPad Prism version 5.03 for Windows. When cell lines from different patients and controls (three LSFC and four controls) were included in the experiment, a linear hierarchical model was built to take into account the different sources of variance. In order to test for a difference between patients and controls, the test statistics were based on the variance between cell lines. These statistical analyses were performed with ‘R’ statistical programming environment (version 3.1.1; http://www.r-project.org/). A *p* < 0.05 was considered statistically significant.

## Results

### Mitochondrial functional phenotype in LSFC fibroblasts

**Fig 1:**
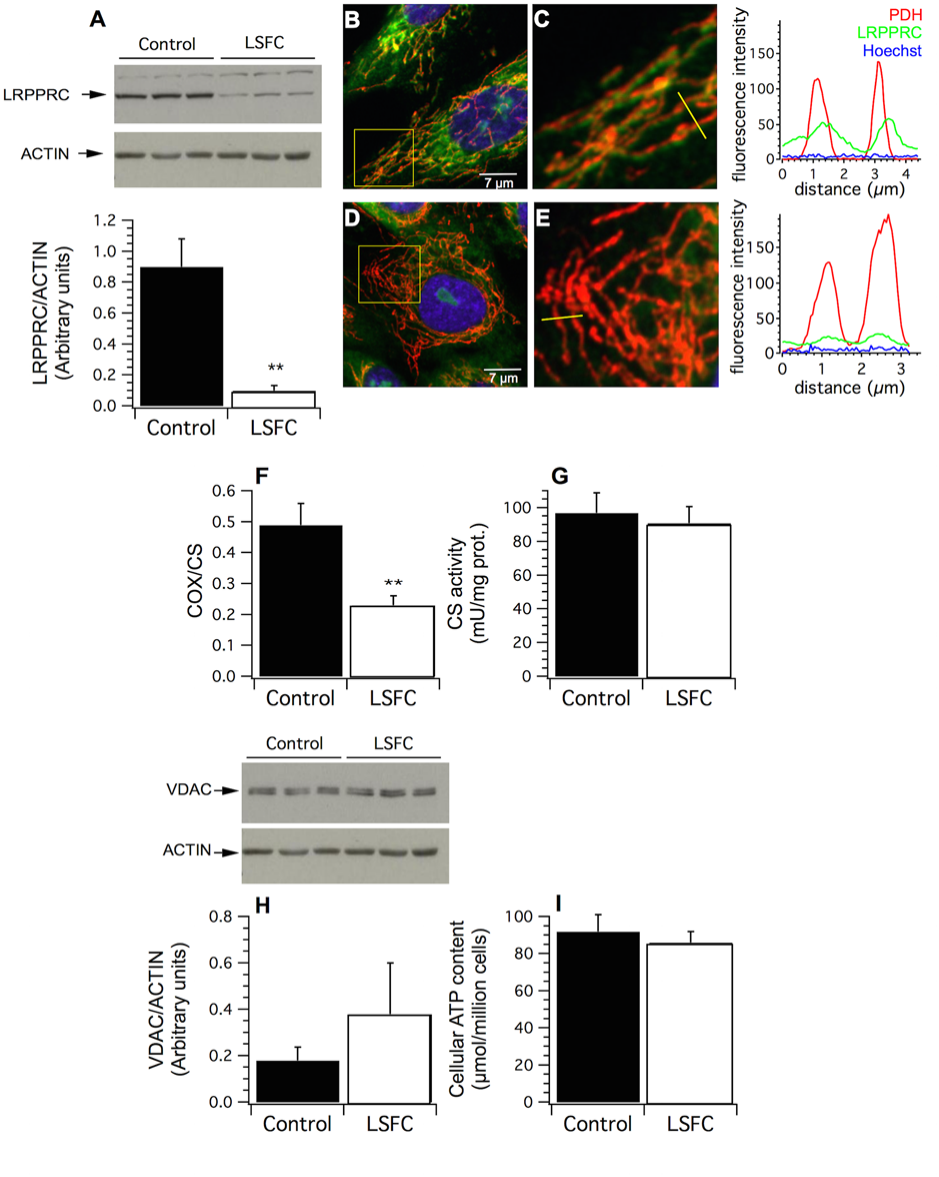
Effect of the LRPPRC A354V mutation on LRPPRC content, mitochondrial content and cellular ATP levels. **(A)** Immunoblot and densitometric analysis of LRPPRC content in whole cell lysates from control and LSFC fibroblasts (n = 4). Representative immunofluorescence images of control **(B;C)** and LSFC fibroblasts **(D;E)** labeled with anti-LRPPRC (green) and anti-pyruvate dehydrogenase (red) antibodies. Overlay images (yellow), and line scan analysis show the cellular distribution of LRPPRC in the mitochondrial compartment. **(F)** Cytochrome c oxidase (COX) enzyme activity in whole cell lysates from control and LSFC fibroblasts was normalized to that of the mitochondrial marker citrate synthase (CS) to take into account possible differences in mitochondrial content (n = 4). **(G)** Immunoblot and densitometric analysis of VDAC, a mitochondrial marker protein, in whole cell lysates from control and LSFC fibroblasts (n = 3). **(H)** Citrate synthase activity in whole cell lysates from control and LSFC fibroblasts (n = 4). **(I)** Cellular ATP content in control and LSFC fibroblasts (n = 4). Data are expressed as means ± S.E. Experiments were performed in one control (EBS-4) and one LSFC cell line (AL-006). Difference between control and LSFC cells was assessed with a paired t-test. ** Significantly different from the control group *p* ≤ 0.01. Statistical power for LRPPRC and COX was 97%.

Consistent with previous results, steady-state levels of LRPPRC immunoreactivity was reduced to less than 30% (*p* = 0.01) of normal values in patients’ fibroblasts (Fig. 1A) [4,5]. Mitochondrial LRPPRC immunofluorescence was also severely reduced in patient cells compared to their control counterparts (Fig. 1B-E). This was accompanied by a 55% reduction in COX enzyme activity, which is also consistent with previous results and is a hallmark of this disease (Fig. 1F, *p* = 0.01). In contrast, CS activity (Fig. 1G) as well as VDAC (Fig. 1H) and HSP60 (S1A Fig.) immunoreactivity, three markers of mitochondrial content, were similar in control and LSFC cells. Furthermore, despite reduced COX activity, ATP content was comparable in control and LSFC fibroblasts, indicating that overall cellular energy status was not impaired under basal conditions (Fig. 1I).

**Fig 2:**
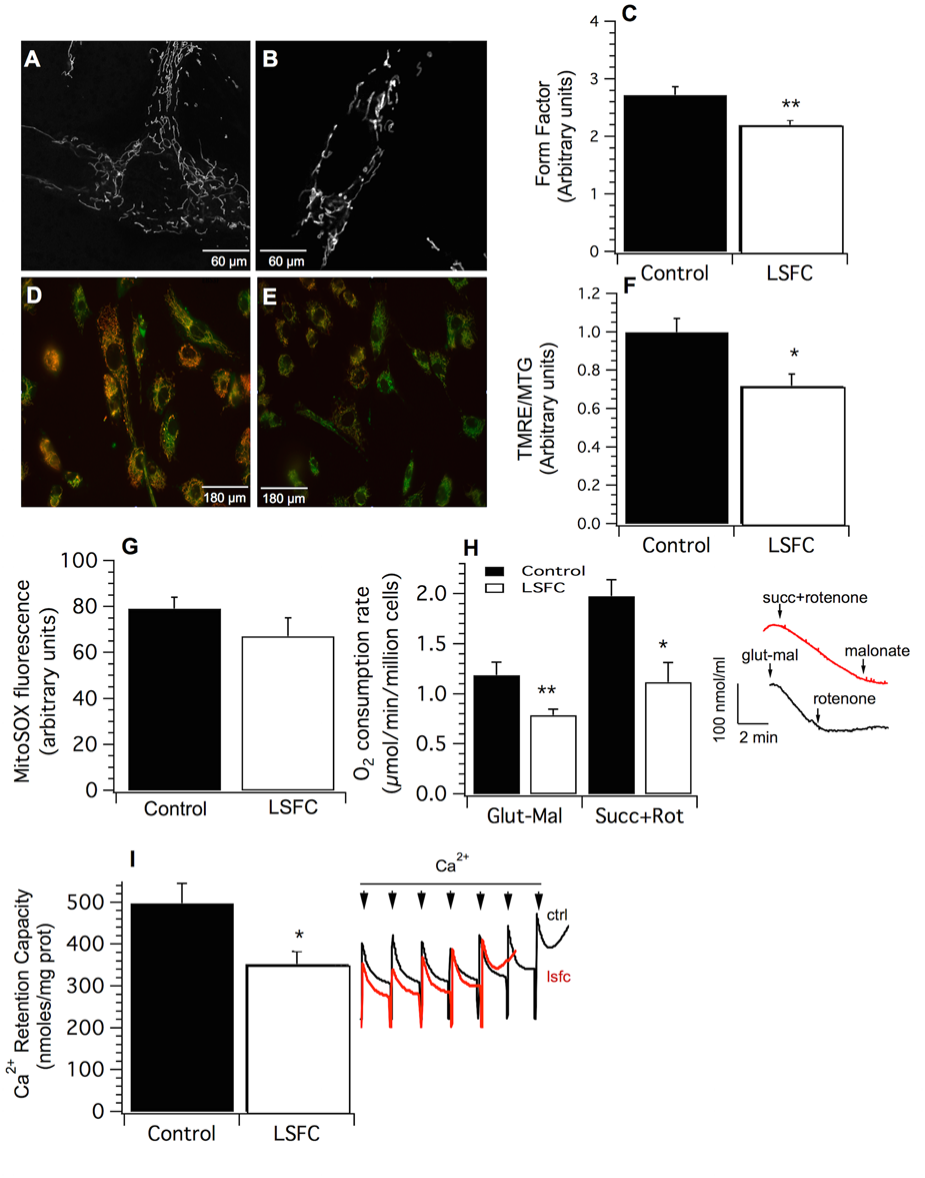
Effect of the LRPPRC A354V mutation on basal mitochondrial network morphology and functions. Representative live cell images of MTG-loaded control **(A)** and LSFC **(B)** fibroblasts used for quantitative analysis of mitochondrial network morphology. **(C)** Form Factor (FF) values calculated using the equation FF = 4π*Area/perimeter2 (n = 6). Representative live cell images of control **(D)** and LSFC **(E)** fibroblasts labeled with TMRE (red) and MTG (green). **(F)** Mitochondrial membrane (ΔΨ) potential expressed as the ratio of TMRE to MTG (n = 5). Lower values are indicative of reduced ΔΨ. **(G)** Mean fluorescence intensity of the mitochondria-specific superoxide probe MitoSOX in control and LSFC fibroblasts (n = 5). **(H)** Maximal ADP-driven respiration in digitonin-permeabilized fibroblasts energized with complex I (5 mM glutamate—2.5 mM malate; Glut-Mal; n = 15) or complex II substrates in presence of the complex I inhibitor rotenone (5 mM succinate + 1 μM rotenone; Succ+Rot; n = 14). Inset shows representative respirometry traces confirming that respiratory rates increased promptly in response to the addition of respiratory substrates, and were potently inhibited by complex I (rotenone), and complex II (malonate) blockers. **(I)** Mitochondrial calcium retention capacity (CRC) in control and LSFC fibroblasts exposed to progressive Ca2+ loading (n = 8). Inset shows representative Ca2+ kinetic tracings observed in control and LSFC fibroblasts. Tracings show progressive Ca2+ accumulation followed by PTP-induced release of accumulated Ca2+. Each spike indicates the addition of a calcium pulse of 83 nmoles. All experiments were performed in one control (EBS-4) and one LSFC (AL-006) cell line, except for the determination of ΔΨ, which was performed in EBS-3 and AL-002. Data are expressed as means ± S.E. Difference between control and LSFC cells was assessed with a paired t-test. Significantly different from the control group: * *p* < 0.05, ** *p* ≤ 0.01. Statistical power: C: 92%; F: 85%; G: 80%; H: Glut-Mal 80%; Succ+Rot: 96%; I: 73%.

Nevertheless, several morphological and functional abnormalities were observed. The mitochondrial network was more fragmented in patient fibroblasts compared to their control counterparts, as shown by reduced FF ratio (Fig. 2A-C, *p* = 0.01), and a trend towards lower AR values (S1B Fig.; *p* = 0.08). Mitochondrial membrane potential was significantly lower in LSFC fibroblasts, as reflected by lower TMRE/MTG ratios (Fig. 2D-F, *p* < 0.05), and a trend towards reduced accumulation of Rh123 in quantitative uptake assays (S1C Fig.; *p* = 0.07). However, superoxide production, measured with the MitoSOX probe, did not differ between control and LSFC fibroblasts under basal conditions (Fig. 2G).

Further experiments were performed in permeabilized cells to characterize respiratory parameters and susceptibility to permeability transition. The latter being a key event in mitochondria-mediated cell death [10,32]. Consistent with the COX defect, maximal ADP-driven oxygen consumption was significantly lower in LSFC fibroblasts compared to their control counterparts (Fig. 2H). This was observed in mitochondria respiring with complex I (glutamate-malate: -35% in LSFC *vs*. Control, *p* = 0.01), and complex II substrates (succinate + rotenone: -43% in LSFC *vs*. Control, *p* < 0.05). In addition, LSFC fibroblasts displayed an increased sensitivity to opening of the PTP when exposed to Ca^2+^, a typical trigger of pore opening (Fig. 2I, *p* < 0.05).

Taken together, these results demonstrate the presence of multiple functional abnormalities in mitochondria from LSFC fibroblasts under baseline conditions. However, these deficiencies were not sufficient to compromise cellular ATP levels.

#### Susceptibility of LSFC fibroblasts to physiopathologically relevant stressors

**Fig 3:**
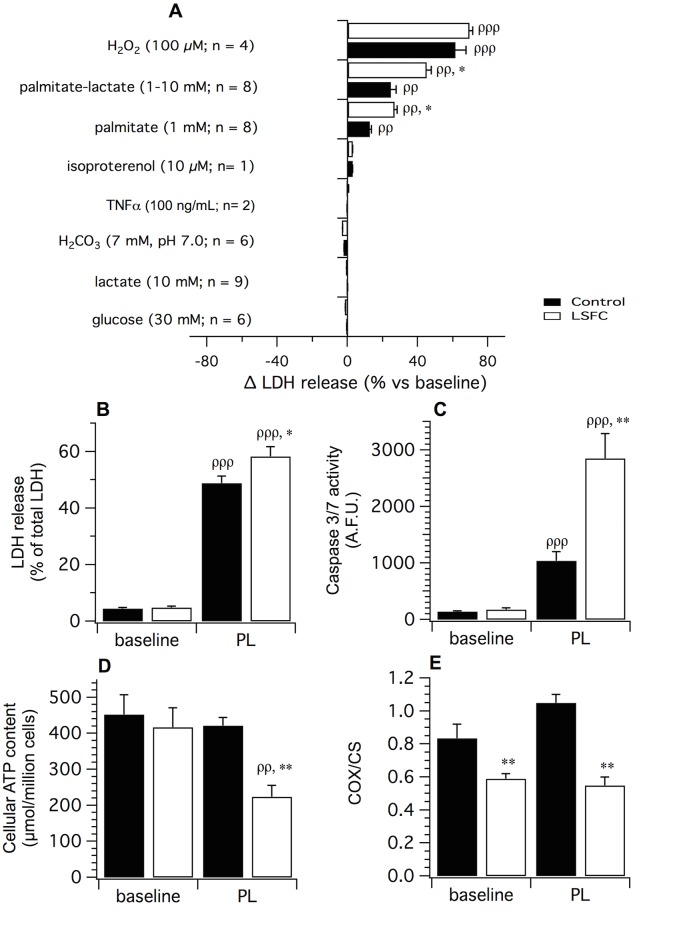
Stress-induced cell death in control and LSFC fibroblasts. **(A)** Lactate dehydrogenase (LDH) release in control and LSFC fibroblasts exposed for 48 h to factors relevant to acidotic crises. **(B)** Mean LDH release (*n* = 5), **(C)** Caspase 3/7 activity (*n* = 7), **(D)** Cellular ATP content (*n* = 7) and **(E)** COX/CS activity ratio (*n* = 3) assessed at baseline and after exposure to PL (palmitate 1 mM; lactate 10 mM) for 34 h (LDH release) or 24 h (other parameters). All experiments were performed in one control (EBS-4) and one LSFC (AL-006) cell line. Data are expressed as means ± S.E. Statistical significance of differences between groups or conditions was assessed with a two-way ANOVA for repeated measures followed by a Bonferroni post hoc test. Significantly different from the control cells in the same experimental condition: * *p* < 0.05, ** *p* < 0.01. Significantly different from baseline within the same experimental group: ^ρ^ *p* < 0.05, ^ρρ^ *p* < 0.01, ^ρρρ^ *p* < 0.001.

Because suboptimal mitochondrial function may predispose to cell death, we next performed experiments to test whether factors relevant to acidotic crises could induce premature cell death of LSFC fibroblasts using LDH release and caspase 3/7 activities as markers. As shown in Fig. 3A, acidosis and high concentrations of glucose and lactate had no significant effect on LDH release and caspase 3/7 activities both in control and patient cells. A similar phenomenon was also observed following exposure to isoproterenol or the inflammatory cytokine TNFα. In contrast, exposure to 1 mM BSA-conjugated palmitate resulted in a significant increase in LDH release in both experimental groups, a phenomenon previously reported in various other cell types (*p* = 0.01) [33–36]. Furthermore, this effect was ~50% greater in LSFC fibroblasts compared to their control counterparts (Fig. 3A; *p* < 0.05). Interestingly, high lactate concentrations enhanced palmitate toxicity by ~1.5 fold (*p* < 0.05), whereas high lactate alone had no effect. As expected, exposure to H_2_O_2_ induced a pronounced increase in cell death in both groups (*p* = 0.001).

The effect of palmitate plus lactate (PL) was further characterized in one control and one patient cell line. As shown in Fig. 3B, PL provoked a significant increase in LDH release (treatment p < 0.001) which was ~25% greater in patient cells compared to their control counterparts (*p* = 0.01). A similar effect was also observed for caspase 3/7 activities (Fig. 3C; *p* = 0.001). This was associated with a 50% reduction in cellular ATP content after a 24 h exposure to PL in LSFC fibroblasts but not in control cells (Fig. 3D; *p* < 0.01). In LSFC fibroblasts, the decrease in ATP content was not related to a further reduction in COX activity beyond the levels observed at baseline (Fig. 3E).

**Fig 4:**
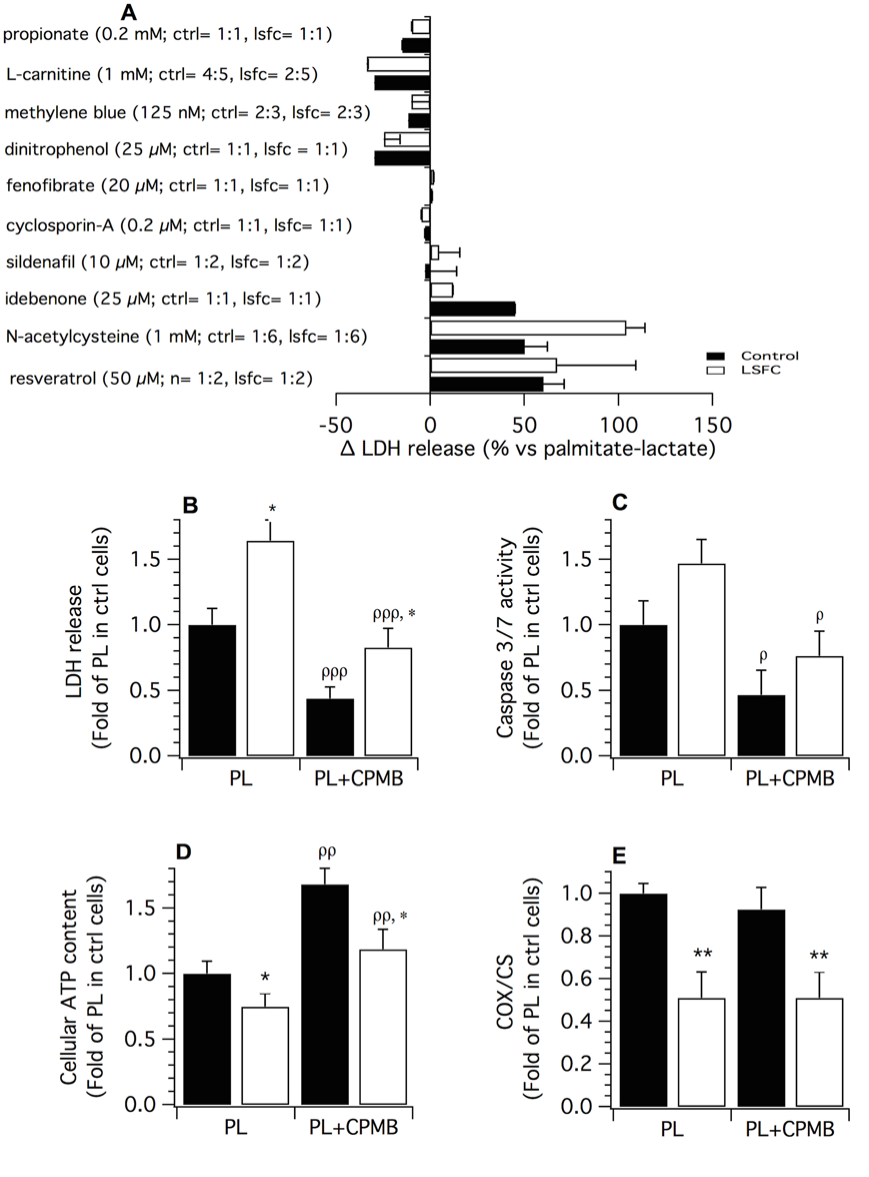
Effect of selected therapeutic agents on palmitate plus lactate (PL)-induced cytotoxicity. **(A)** Lactate dehydrogenase (LDH) release in control and LSFC fibroblasts exposed to PL in presence or absence of therapeutic compounds. The numbers in brackets (ex: control: *n* = 2:6) refer to the number of cell lines tested and of experiments, respectively. **(B)** Mean LDH release (*n* = 5), **(C)** Caspase 3/7 activity (*n* = 5), **(D)** Cellular ATP content (*n* = 7) and **(E)** COX/CS activity ratio (n = 3) measured after exposure to PL (palmitate 1 mM; lactate 10 mM) in absence or presence of CPMB (1 mM L-carnitine; 0.2 mM propionate; 125 nM Methylene blue) for 34 h (LDH release) or 24 h (other parameters). All measurements were performed in 4 control and 3 LSFC cell lines for the aforementioned number of experiments. Data are means ± S.E. and expressed as fold-change relative to the mean value of the PL condition in control (ctrl) cells. Statistical significance of differences between groups and conditions were analyzed with a linear hierarchical model (cf. Methods section). Significantly different from the control cells in the same experimental condition: * *p* < 0.05, ** *p* < 0.01. Significantly different from baseline within the same experimental group: ^ρ^ *p* < 0.05, ^ρρ^ *p* < 0.01, ^ρρρ^ *p* < 0.001.

The effect of PL on LDH release, caspase 3/7 activities, ATP levels and COX activity was then examined in three LSFC and four control cell lines (see Table 1) and similar results were obtained (Fig. 4). The effect of PL on both LDH release and caspase 3/7 activities was also tested in the immortalized cell lines used to characterize baseline mitochondrial function. Although the effect occurred more rapidly in immortalized cells than in primary cells, LSFC fibroblasts still displayed an increased susceptibility to PL-induced cytotoxicity compared to their control counterparts (S2 Fig.).

Overall, these results indicate that LSFC fibroblasts display an enhanced susceptibility to cell death in response to palmitate, either alone or in combination with high lactate that is not associated with further deterioration of COX activity.

### Therapeutic interventions to reduce palmitate plus lactate-induced cell death

To identify treatment strategies, and gain insights on the mechanisms underlying cytotoxicity in response to PL, control and patient cells were exposed to therapeutic agents selected based on their known effects on mitochondrial function. As shown in Fig. 4A, the anti-oxidant compounds idebenone, N-acetyl cysteine, and resveratrol, all exacerbated PL-induced LDH release, whereas the PPARα agonist fenofibrate, the cyclophilin-D ligand cyclosporin-A and the phosphodiesterase-5 inhibitor sildenafil, had no effect on this parameter. In contrast, L-carnitine, propionate, and methylene blue reduced PL-induced LDH release by 15–30%. Interestingly, low concentrations of the uncoupler dinitrophenol also provided protection against the cytocoxic effects of PL. However, these experiments were not pursued further due to the cytotoxicity of dinitrophenol at higher concentrations.

The protective effect of the combined administration of L-carnitine, propionate and methylene blue (CPMB) was then tested in four control and three patient cell lines because of its potential greater physiopathological relevance. As shown in Fig. 4B, CPMB reduced LDH release induced by PL by ~45% both in control and patient cells (*p* = 0.001). It should be noted, however, that LDH release remained higher in patient cells compared to their control counterparts (*p* < 0.05). CPMB also reduced caspase 3/7 activity by ~50% in both control and LSC cell lines compared to values observed in the presence of PL alone (Fig. 4C; *p* < 0.05). Administration of CPMB also increased cellular ATP content 1.6–1.7 fold above values observed in cells exposed to PL alone (Fig. 4D; *p* = 0.01). As shown in Fig. 4E, the protective effect of CPMB was not associated with improved COX activity.

Overall, these results thus indicate that CPMB provides an additive protection against the cytotoxic effect of PL, while compounds acting on the PTP and antioxidants either have no effect or enhance cytotoxicity, respectively.

## Discussion

Recent studies have led to significant advances in our understanding of the molecular mechanisms linking the LRPPRC mutations to COX deficiency in LSFC patients [4–7]. Yet, it remains unclear how this biochemical defect brings about the pathological phenotype associated with LSFC, due in part to a lack of data on the functional impact of LRPPRC gene mutation, and our limited knowledge of factors triggering fatal lactic acidosis crises. In the present study, we report that mitochondria from LSFC fibroblasts present several morphological and functional abnormalities that do not compromise cellular energy homeostasis under basal conditions, as evidenced by normal cellular ATP levels, but may contribute to cellular vulnerability in response to stress. We also provide evidence that fibroblasts derived from LSFC patients display enhanced susceptibility to cell death when exposed to palmitate, a phenomenon that is exacerbated by high lactate concentration. Furthermore, screening with a range of pharmacological compounds acting on various facets of mitochondrial function has provided insights into the mechanisms potentially contributing to this nutrient-induced cytotoxicity, and identified L-carnitine, propionate and methylene blue either alone or as a combined intervention that effectively reduces cell death in these conditions.

### Mitochondrial phenotyping reveals multiple dysfunctions associated with LRPPRC A354V mutation

Our understanding of the role of LRPPRC on mitochondrial function is scarce, and derived primarily from studies in which wild type LRPPRC was knocked-down in a cancer or liver transformed cell line [37,38]. Previous studies by our groups and others have examined the protein abundance of the various ETC complexes in fibroblasts obtained from several LSFC patients, including our patients, and consistently reported only a decrease in COX abundance [1,4]. A generalized assembly defect in other ETC complexes in LSFC fibroblasts was observed only after siRNA-mediated knockdown of LRPPRC [4]. Consistent with the decreased COX protein abundance in LSFC fibroblasts, COX activity was also decreased by ~50%. To the best of our knowledge the activity of other complexes has not been measured. In this context, one of the objectives of the present study was to determine the consequences of the LRPPRC A354V mutation on various facets of mitochondrial function using LSFC fibroblasts to better understand cellular physiopathology. At the level of respiratory performance, our results indicate that in LSFC fibroblasts, reduced expression of LRPPRC and the resulting 55% loss in COX activity was sufficient to reduce maximal ADP-stimulated respiration by 35% when the ETC was energized with substrates for complex I, and by 43% when mitochondria respired with substrates for complex II. These results suggest that in brain and liver cells, in which COX deficiency is greater (*i*.*e*. 80% loss of activity) than in fibroblasts [1,4], the impairment of OXPHOS capacity is likely greater, putting these organs at greater risk of energetic crises when ATP demand rises.

In addition to reduced OXPHOS capacity, we also observed that, compared to their control counterparts, mitochondria of LSFC fibroblasts maintained a lower membrane potential under basal conditions, concurring with observations in LRPPRC knock-down HeLa cells [38]. Similar results were also reported in fibroblasts from *Surf1* patients, which present a more severe COX defect than LSFC fibroblasts [39], and from patients with complex I deficiencies related to mutations of nuclear-encoded genes [40,41]. The magnitude of the depolarization observed in our study and others [39–41] is sufficient to impair mitochondrial functions such as ATP synthesis, and metabolite/ion transport, which are very sensitive to small changes in ΔΨ [42]. In the present study, we did not find any difference in baseline cellular ATP content between control and LSFC fibroblasts. This may reflect enhanced glycolytic ATP production, which could compensate for any reduction in OXPHOS. Thus, we cannot exclude the possibility of a subtle impairment in mitochondrial ATP synthesis, as previously demonstrated in fibroblasts from complex I-deficient patients using more direct approaches such as mitochondria-targeted luciferase [40,41].

Increased production of superoxide anion (O_2_^-•^-) is a common consequence of respiratory chain inhibition, and is thus frequently implicated as a pathogenic mechanism in genetic mitochondrial diseases [40,43]. However, in the present study, we found no signs of increased mitochondrial O_2_^-•^ production in LSFC fibroblasts. This result contrasts with the substantial increase in mitochondrial O_2_^-•^ production frequently, [40,41,44,45,46] albeit not systematically [47], observed in human and rodent cells harboring genetic complex I and complex III defects. Such a difference may be related to the fact that, in these latter cases, mutations are affecting the two complexes (*i*.*e*. CI and CIII) that are directly responsible for O_2_^-•^ formation, whereas COX does not release O_2_^-•^ *per se* and only modulates its formation indirectly by affecting the redox state of the upstream ETC complexes [48]. Interestingly, Diaz et al. recently reported that complex III-deficient RISP-knockout mice show early and severe ROS damage that is observed only at a later stage in pathology progression in COX10-deficient mice [46]. Together these results support the notion that the contribution of oxidative stress is disease-specific and may be less prominent in cases of isolated COX defects such as in LSFC.

While ROS production was not significantly altered in LSFC fibroblasts, we found their mitochondria to be significantly more susceptible to opening of the PTP when challenged with Ca^2+^. This suggests that cells could be more susceptible to activation of cell death when faced with pathological conditions where cellular Ca^2+^ homeostasis is altered. In the brain, this typically occurs during stroke episodes [49], which commonly occur in LSFC patients and contribute to neuronal death during acidotic crises [3]. The mechanisms responsible for this increased susceptibility to Ca^2+^-induced permeability transition were not investigated in depth in the present study. However, it is interesting to note that partial depolarization, as observed in LSFC cells, is a key sensitizer to Ca^2+^-induced opening of the PTP [50], which could explain at least in part their reduced tolerance to Ca^2+^.

Increasing evidence suggests that alterations in mitochondrial morphology are both a cause and a consequence of changes in mitochondrial function (reviewed in [51]). In general, studies involving genetic knockdown or overexpression of fusion and fission proteins have shown that increased fragmentation associates with depolarization, reduced OXPHOS efficiency, enhanced ROS production, and/or propensity to permeability transition and cell death, which has led to the suggestion that network fragmentation is a general reflection of mitochondrial and cellular stress [51]. Based on the available evidence, we therefore believe that increased fragmentation of the network observed in LSFC fibroblasts, which is comparable in magnitude to that observed in fibroblasts from patients with other genetic respiratory chain defects [31,52,53], is indicative of prevailing mitochondrial stress. Interestingly, recent studies in fibroblasts from mice and patients with isolated complex I deficiencies have suggested a close link between network fragmentation and increased O_2_^-•^ production [31,54]. Although this explanation may hold true for complex I defects, our results indicate that this is unlikely the case in LSFC fibroblasts. Reduced membrane potential, which is a key trigger of mitochondrial fission, could however play a role [51,55].

### LRPPRC A354V mutation increases susceptibility to palmitate plus lactate-induced cell death

In LSFC patients, fulminant acidotic crises reflect the transition from compensated COX deficiency to profound mitochondrial and cellular dysfunctions, which ultimately lead to cell death, and irreversible organ failure. Although cultured fibroblasts are limited with respect to translatability to the *in vivo* situation, they currently constitute the only model to investigate conditions that may promote this transition in LSFC patients, and to test potential cytoprotective approaches.

In the present study, we found most conditions tested individually to be innocuous to LSFC (and control) fibroblasts, including exposure to high glucose, high lactate, low pH, isoproterenol, and TNFα. However, we found LSFC fibroblasts to be significantly more susceptible to the cytotoxic effect of palmitate given alone at physiologically relevant concentrations, or in combination with high lactate. To our knowledge, enhanced sensitivity to palmitate-induced cell death has not been reported previously in cells from patients with mitochondrial diseases. However, parallels can be made with the adverse effects of high fat diets in some types of mitochondrial pathologies [56–58] and with clinical observations that nutrient excess can precede acidotic crises in LSFC patients [3]. Our results thus demonstrate that impaired ETC function can limit the ability of cells to handle excess substrate supply, which in the case of saturated long-chain fatty acids, has well-known cytotoxic consequences. It should be noted, however, that positive effects of high-fat diets have been observed in patient fibroblasts and the Harlequin mouse model of complex I deficiency [59]. These conflicting results thus highlight the necessity of taking into account the nature of the biochemical defect before considering the use of high-fat diets for patient management.

Studies in several cell types have previously linked the toxicity of palmitate to various factors including accumulation of toxic high molecular weight lipid metabolites such as ceramides [33] and mitochondrial functional alterations caused by increased free fatty acid supply [33,60–62]. A COX deficiency could thus exacerbate one or several of these abnormalities in LSFC cells, resulting in enhanced cellular dysfunction and death. While our aim was not to characterize these mechanisms in detail, our screening with therapeutic compounds targeting various aspects of mitochondrial functions provides interesting insights.

### Protective strategies against palmitate plus lactate-induced cytotoxicity

A key observation in the present study is that compounds with a cytoprotective effect all shared common features that were clearly distinct from those that either had no effect or were detrimental to cell survival. More specifically, we found that all protective compounds promote fatty acid and mitochondrial metabolism. Indeed, propionate is an anaplerotic substrate that provides Krebs cycle intermediates at the level of succinyl-CoA, which is upstream of a substrate level phosphorylation reaction. Krebs cycle intermediates are essential to sustain efficient oxidation of acetyl-CoA derived from β-oxidation, and limit accumulation of toxic lipid by-products [25,63]. Similarly, L-carnitine stimulates long-chain acyl-CoA transport within the mitochondria, which can limit the accumulation of fatty acids in the cytoplasmic compartment [25,63].

Strikingly, we observed that nearly all the protective compounds also promote electron flux in the ETC independent of ATP synthesis. Indeed, by stimulating acyl-CoA transport, which depletes the mitochondrial electrochemical gradient, L-carnitine can exert a mild uncoupling effect [64]. Such mild uncoupling is clearly protective against PL-induced toxicity, as demonstrated in the present study by the beneficial effect of a low dose of the uncoupler dinitrophenol. As for methylene blue, it is a well-known redox-active agent that cycles readily between oxidized (MB) and reduced (MBH_2_) forms using various redox centers present in mitochondria and other cellular compartments [65]. In mitochondria, methylene blue directly oxidizes complex I to form MBH_2_ which is then re-oxidized by cytochrome *c* and COX, or directly by O_2_ in cases where COX activity is low, such as in LSFC fibroblasts [65]. Similar to mild uncouplers, methylene blue thus promotes electron flux in the ETC, and lowers the mitochondrial redox state, which is high in conditions of excess nutrients [66], particularly in cells with an impaired ETC.

It is well established that at high mitochondrial redox states, ROS production from complex I and III is significantly higher, which can induce oxidative damage and trigger cell death [48]. In addition, because NADH levels are increased, multiple cellular processes that depend on the availability of its oxidized counterpart, NAD^+^, can be impaired, which contributes to a state of reductive stress [66,67]. Interestingly, in our screening experiments, we observed that antioxidant compounds (*i*.*e*. idebenone, N-acetyl cysteine and resveratrol), rather than being protective, systematically worsened PL-induced toxicity. This observation, which is in line with previous observations, highlights the limited and sometimes harmful effect of antioxidants in patients with genetic mitochondrial disease [68] and suggests that oxidative damage does not play a role in the increased susceptibility of LSFC fibroblasts to PL-induced cell death. In fact, because anti-oxidant treatments tend to increase the reduced state of endogenous redox couples, these data further support to the notion that reductive stress could be the main culprit. It should also be noted that methylene blue was reported to increase COX content, presumably through redox regulation of transcriptional activity [13,65], which could have been beneficial to LSFC cells. However, we did not observe this effect in the present study, as evidenced by the absence of changes of the *in vitro* COX enzyme activity.

Previous studies reported that palmitate induces cell death through permeabilization of mitochondrial membrane involving PTP-dependent and-independent mechanisms [33,35]. Since LSFC fibroblasts displayed increased sensitivity to Ca^2+^-induced opening of the PTP, we reasoned that enhanced permeability transition could also partly underlie the susceptibility of LSFC fibroblasts to PL. However, results from our screening experiments failed to show any beneficial effect of the PTP inhibitor cyclosporin-A both in control and LSFC fibroblasts, indicating that permeability transition likely played a negligible role in these conditions. Similar results were also obtained with sildenafil, which was previously shown to inhibit stress-induced opening of the PTP [19,69].

## Conclusion

In conclusion, results from our study provide the first comprehensive assessment of the mitochondrial function phenotype in fibroblasts from LSFC patients. We show that the LRPPRC A354V mutation, and resulting COX deficiency, is associated with several morphological and functional mitochondrial abnormalities. These alterations include an increased fragmentation of the mitochondrial network, a hallmark of the mitochondrial stress response [51], impaired OXPHOS capacity, lower membrane potential, and increased sensitivity to Ca^2+^-induced permeability transition. However, in contrast to isolated complex I [40,41,44,45] and complex III [46] deficiencies, mitochondrial superoxide production is not enhanced in LSFC cells, which supports the notion that the pathogenic importance of oxidative stress is disease-specific [46]. Our results also demonstrate that LSFC fibroblasts are more susceptible to the cytotoxic effect of palmitate alone or in combination with lactate. This phenomenon could be linked to an impaired ability to oxidize excess fatty acids and mitochondrial reductive stress. Collectively, our results raise some concerns about the potential negative impact of a high-fat diet and of antioxidant treatments in LSFC patients. Although there are clear limitations related to extrapolation of data obtained in fibroblasts *in vitro* to the *in vivo* situation, we believe that these data provide important new information that should be validated once murine models of LSFC become available.

## Supporting Information

S1 Fig Effect of the LRPPRC A354V mutation on HSP60 content, basal mitochondrial network and membrane potential. **(A)** Immunoblot of HSP60 content in whole cell lysates from control and LSFC fibroblasts (n = 2). **(B)** Quantitative analysis of mitochondrial network morphology. The Aspect Ratio value was calculated using the following equation: long axis/short axis (n = 6). **(C)** Mitochondrial membrane (ΔΨ) potential was examined using Rhodamine 123 uptake (Rh123; n = 3) Cellular uptake of Rh123 was calculated from a standard curve generated using known concentrations of Rh123. All experiments were performed in one control (EBS-4) and one LSFC (AL-006) cell line.
(TIFF)

S2 Fig Palmitate plus lactate (LP)-induced lactate dehydrogenase (LDH) release in primary and immortalized fibroblast cells. **(A)** Time course of LDH release from primary and immortalized control fibroblasts exposed to 1 mM palmitate and 10 mM lactate (*n* = 3). **(B)** LDH release in primary control and LSFC fibroblasts exposed to PL (n = 4). **(C)** LDH release in immortalized control and LSFC fibroblasts exposed to PL (n = 3). All experiments were performed in one control (EBS-4) and one LSFC (AL-006) cell line. *p < 0.05
(TIFF)
