# Supplementary figures and images for: Mitochondrial Vulnerability and Increased Susceptibility to Nutrient-Induced Cytotoxicity in Fibroblasts from Leigh Syndrome French Canadian Patients
Source: PLoS One. 2015 Apr 2;10(4):e0120767. doi: 10.1371/journal.pone.0120767 (PMC4383560; doi:10.1371/journal.pone.0120767)

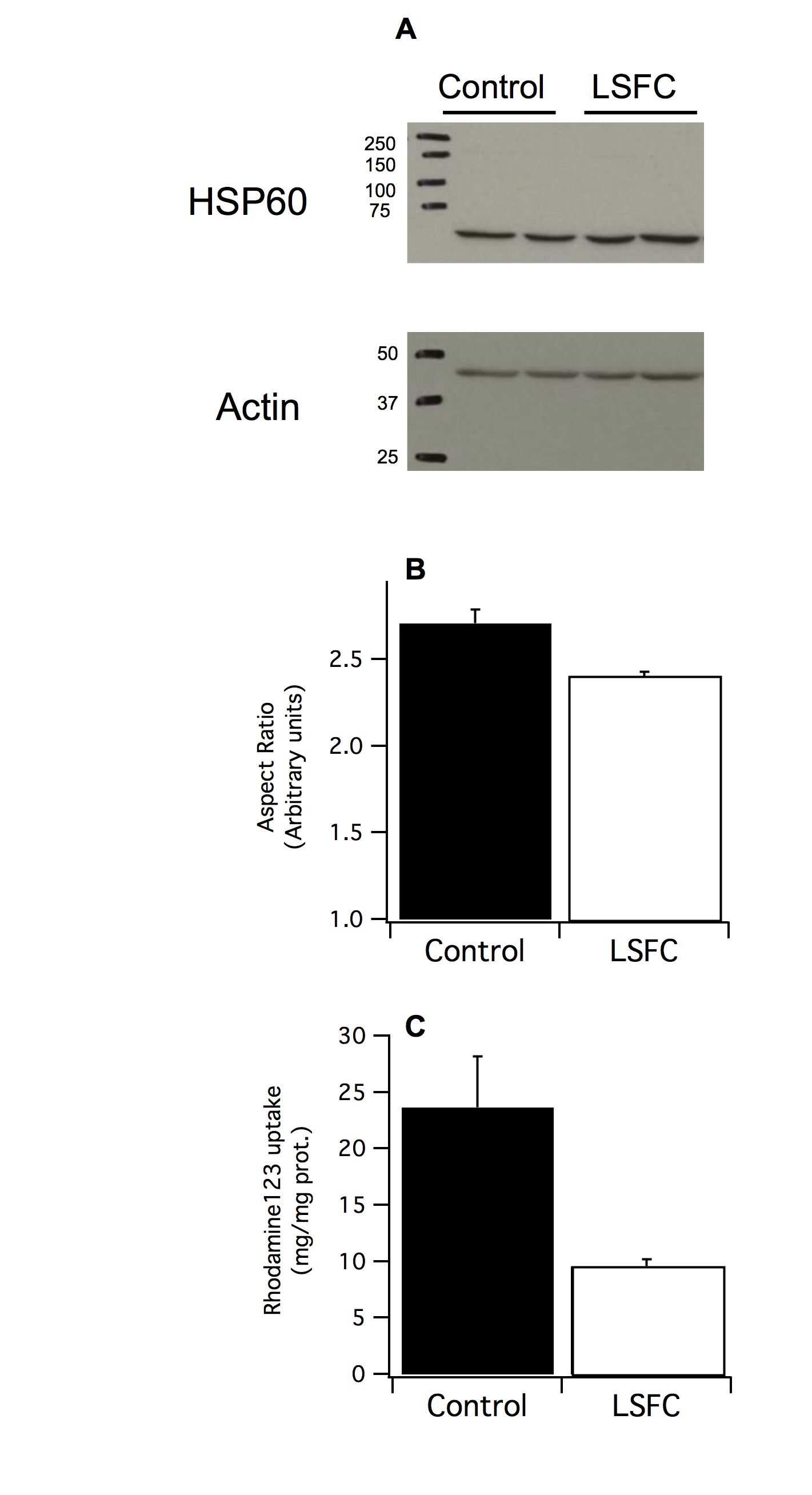

Supplement: S1 Fig — (A) Immunoblot of HSP60 content in whole cell lysates from control and LSFC fibroblasts (n = 2). (B) Quantitative analysis of mitochondrial network morphology. The Aspect Ratio value was calculated using the following equation: long axis/short axis (n = 6). (C) Mitochondrial membrane (ΔΨ) potential was examined using Rhodamine 123 uptake (Rh123; n = 3) Cellular uptake of Rh123 was calculated from a standard curve generated using known concentrations of Rh123. All experiments were performed in one control (EBS-4) and one LSFC (AL-006) cell line. (TIFF) [file pone.0120767.s001.tiff]

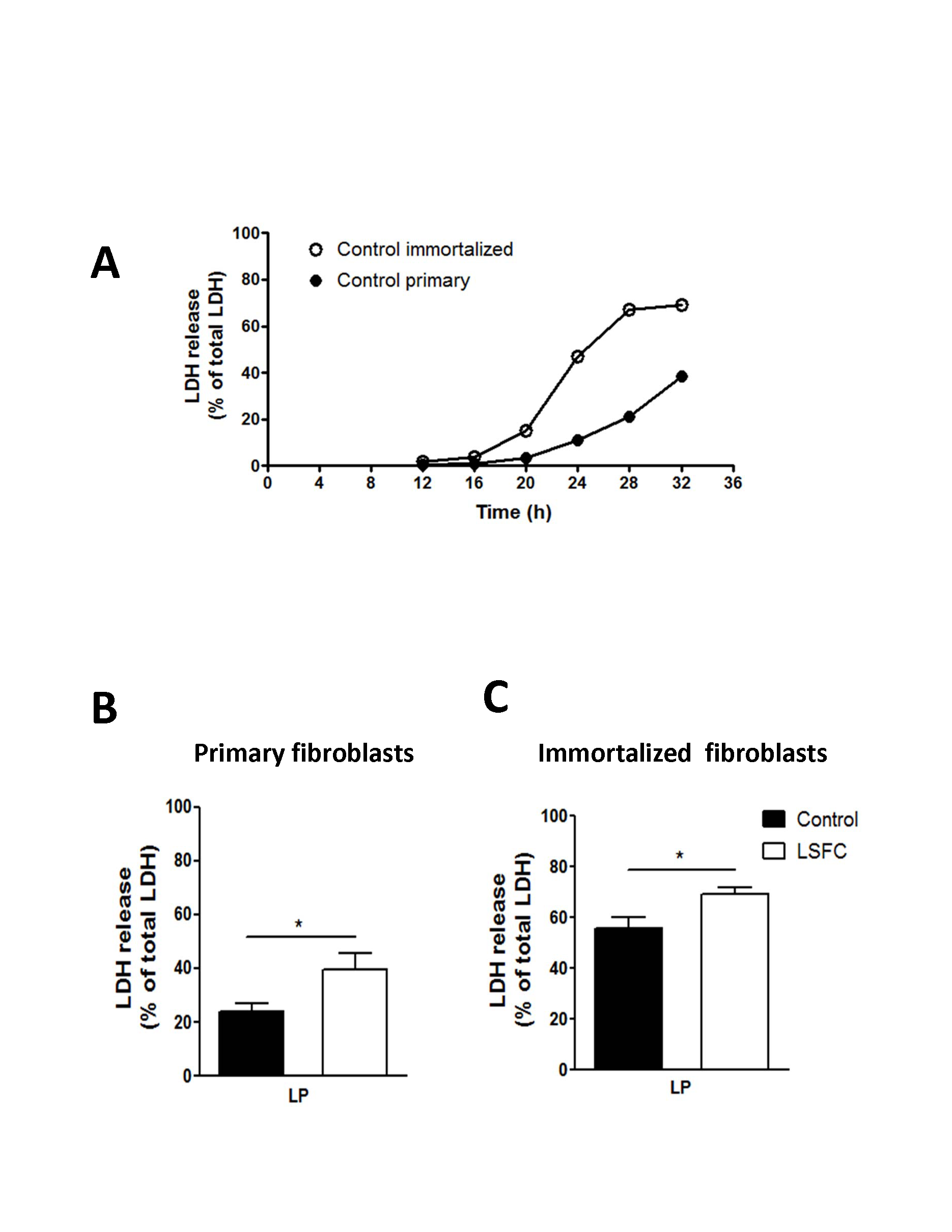

Supplement: S2 Fig — (A) Time course of LDH release from primary and immortalized control fibroblasts exposed to 1 mM palmitate and 10 mM lactate (n = 3). (B) LDH release in primary control and LSFC fibroblasts exposed to PL (n = 4). (C) LDH release in immortalized control and LSFC fibroblasts exposed to PL (n = 3). All experiments were performed in one control (EBS-4) and one LSFC (AL-006) cell line. *p < 0.05 (TIFF) [file pone.0120767.s002.tiff]
